# Myelin oligodendrocyte glycoprotein reactive Th17 cells drive Janus Kinase 1 dependent transcriptional reprogramming in astrocytes and alter cell surface cytokine receptor profiles during experimental autoimmune encephalomyelitis

**DOI:** 10.1038/s41598-024-63877-0

**Published:** 2024-06-07

**Authors:** Sarah M. Milne, Anirudhya Lahiri, Cristina L. Sanchez, Micah J. Marshall, Ishrat Jahan, Gordon P. Meares

**Affiliations:** 1 Department of Microbiology, Immunology, and Cell Biology, West Virginia University, Morgantown, WV 26506 USA; 2 Department of Neurology, The Ohio State University College of Medicine, IBMR 415D, 460 Medical Center Drive, Columbus, OH 43210 USA; 3 Department of Neuroscience, West Virginia University, Morgantown, WV 26506 USA; 4 Rockefeller Neuroscience Institute, Morgantown, WV 26506 USA

**Keywords:** Glial biology, Neuroimmunology, Multiple sclerosis

## Abstract

Multiple sclerosis (MS) is an autoimmune demyelinating disease affecting the central nervous system (CNS). T helper (Th) 17 cells are involved in the pathogenesis of MS and its animal model of experimental autoimmune encephalomyelitis (EAE) by infiltrating the CNS and producing effector molecules that engage resident glial cells. Among these glial cells, astrocytes have a central role in coordinating inflammatory processes by responding to cytokines and chemokines released by Th17 cells. In this study, we examined the impact of pathogenic Th17 cells on astrocytes in vitro and in vivo. We identified that Th17 cells reprogram astrocytes by driving transcriptomic changes partly through a Janus Kinase (JAK)1-dependent mechanism, which included increased chemokines, interferon-inducible genes, and cytokine receptors. In vivo, we observed a region-specific heterogeneity in the expression of cell surface cytokine receptors on astrocytes, including those for IFN-γ, IL-1, TNF-α, IL-17, TGFβ, and IL-10. Additionally, these receptors were dynamically regulated during EAE induced by adoptive transfer of myelin-reactive Th17 cells. This study overall provides evidence of Th17 cell reprogramming of astrocytes, which may drive changes in the astrocytic responsiveness to cytokines during autoimmune neuroinflammation.

## Introduction

The pathogenesis of multiple sclerosis (MS) and the widely used animal model of experimental autoimmune encephalomyelitis (EAE) involves central nervous system (CNS) infiltration of immune cells including autoreactive T cells, B cells, monocytes, and neutrophils^1–4^. These autoreactive T and B cells respond to CNS antigens leading to inflammation, demyelination, neuronal and axonal damage^5,6^. In EAE, these processes are recapitulated by immunization with myelin antigens such as myelin oligodendrocyte glycoprotein (MOG) or by adoptive transfer of encephalitogenic T cells^7,8^. Following immunization-induced priming, myelin antigen-specific T cells are activated, proliferate, and differentiate into effector T cells. These effector T cells can enter the CNS and produce pro-inflammatory cytokines that drive neuroinflammation^5^. Following receptor ligation, these cytokines evoke transcriptional changes predominantly through Janus kinase (JAK)/signal transducer and activator of transcription (STAT) and nuclear factor (NF)-κB dependent mechanisms^9,10^. Targeting these pathways has been shown to be protective in models of neuroinflammation^10–12^. Infiltration of immune cells into the CNS not only impacts oligodendrocytes but also drives alterations in the functional state of microglia and astrocytes^13–15^. Ultimately, the inflamed CNS tissue is a complex milieu of cellular players and soluble mediators that promote both pathology and resolution. In MS, the pathology-inducing mechanisms overwhelm the inflammation resolving and pro-reparative processes^6,16,17^.

There are a variety of CD4 + T helper (Th) cell subtypes that include Th1, Th2, Th17, Th9, T regulatory cells, and follicular helper T cells, each with unique and important functions within the immune system. Myelin reactive T cells, including Th17 and Th1 cells, can causally drive EAE and are implicated in MS pathogenesis^14,18^. Th17 cells are identified by the cell surface markers cluster of differentiation (CD)161, chemokine receptor (CCR)6, CCR4, interleukin receptor (IL)-23R, and IL-1R, the lineage-defining transcription factor retinoic-acid-receptor-related orphan nuclear receptor (ROR)γτ, and they are producers of IL-17A-F, IL-21, and IL-22^19–21^. Physiologically, Th17 cells have been characterized by their ability to respond against extracellular pathogens and fungi^22^. However, pathogenic Th17 cells have been identified as potent inducers of EAE and produce IL-17, granulocyte–macrophage colony stimulating factor (GM-CSF), and interferon (IFN)-γ^23–25^. Numerous studies present evidence of T cell-astrocyte interactions in MS and EAE and these interactions ameliorate or worsen inflammatory responses within the CNS depending on the T cell subtype involved^14,26^. In MS and EAE CD4 + T cells disrupt the blood brain barrier (BBB) and release inflammatory mediators that modify the function of resident glial cells^14^. Under neuroinflammatory conditions astrocytes undergo morphological and functional changes inducing a reactive phenotype^13^. Both Th1 and Th17 effector molecules can activate astrocytes and increase pro-inflammatory responses such as gliosis, recruitment of microglia, and inducing additional migration of Th17 cells^14^. In response to inflammatory stimuli, astrocytes produce several T cell recruiting chemokines such as CC chemokine ligand (CCL)2, CCL20, CCL5, C-X-C motif chemokine ligand (CXCL)1, and CXCL10^27–31^.

Previous EAE studies have shown that astrocytes serve both protective and detrimental roles in facilitating or limiting inflammation, tissue damage, and repair^32,33^. Additionally, using adoptive transfer of myelin specific CD4 T cells, it has been demonstrated that T cells can directly influence astrocytes. Similarly, astrocytes can modulate the activity and polarization of T cells^34^. Astrocytes respond to and produce cytokines and thereby influences the neuroinflammatory environment^17,35–39^. Astrocytes have important roles in maintaining CNS homeostasis which include formation of the BBB, supporting neurons, and aiding in synapse formation. Recent transcriptomic studies have shown that astrocytes have regional heterogeneity, alluding to functional diversity ^40–47^. Similarly, through differential use of transcriptional regulators, astrocytes mount disease selective responses^48^. Astrocytes undergo modifications in form and function that vary by severity of the injury and through gain and loss of functions. During EAE astrocytes show transcriptomic diversity both regionally and based on disease severity^40^. In neuroinflammation cytokines and chemokines are central molecules shaping the response and influencing outcome. A variety of pro- and anti-inflammatory cytokines have been shown to influence astrocyte function; however, the number, distribution, and regulation of the cognate receptors has not been well defined.

Astrocytic responses to cytokines have implications on disease. Previous studies have shown that IFN-γ appears to have both pro- and anti-inflammatory effects in EAE, which is dependent on disease state^49–51^. IFN-γ signaling in astrocytes has been shown to upregulate expression of adhesion molecules, ICAM-1 and VCAM, promoting T cell entry into the CNS and direct localization of T cells during EAE^50,52^. Similarly, tumor necrosis factor (TNF)-α signaling through TNFR1 stimulates astrocytic VCAM expression that facilitates entry of encephalitogenic T cells^53^. Astrocytes also respond to and produce anti-inflammatory cytokines including transforming growth factor (TGF)β and IL-10. However, TGFβ signaling in astrocytes during autoimmune neuroinflammation may contribute to disease progression^54–56^. Furthermore, it has been demonstrated that EAE is more severe in IL-10 deficient mice^57,58^. Consistent with this anti-inflammatory role, there is an augmented neuroinflammatory response to peripheral lipopolysaccharide when the IL-10 receptor is deleted from astrocytes^59,60^.

A wealth of evidence supports the premise that astrocytes are reprogrammed by infiltrating immune cells. However, the direct response of astrocytes to encephalitogenic Th17 cells and the potential changes in cytokine responsiveness across EAE disease course have not been fully defined. In this study we examined the astrocytic response to pathogenic Th17 cells in vitro and in vivo. The data suggest that Th17 cells drive widespread transcriptomic changes in astrocytes that are, in part, through a JAK1-dependent mechanism. Additionally, we identified that cell surface expression of key cytokine receptors including those for IFN-γ, IL-1, TNF-α, IL-17, TGFβ, and IL-10 vary across different CNS regions and during EAE.

## Results

### MOG-reactive Th17 cells produce JAK/STAT activating cytokines

**Figure 1:**
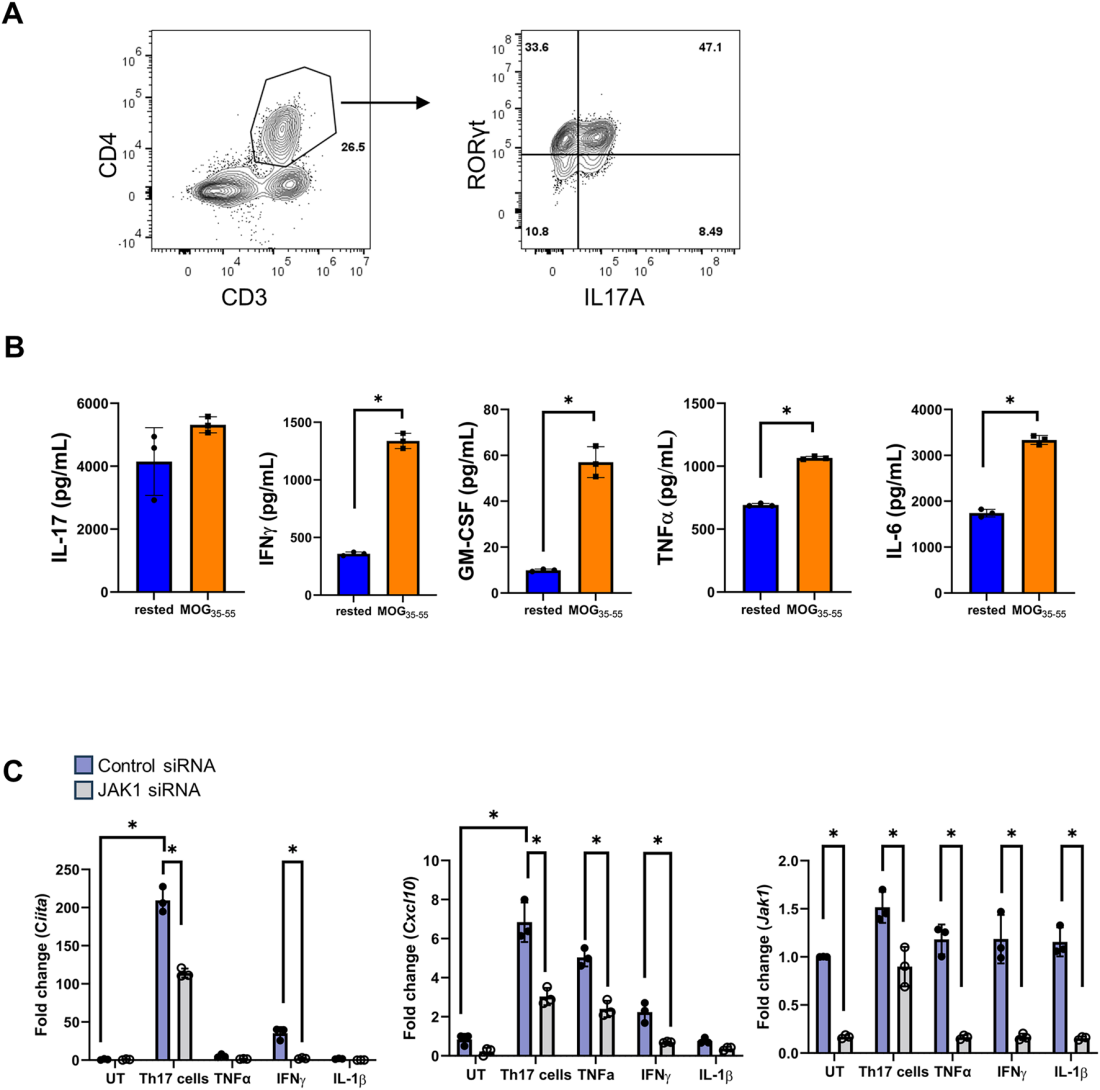
MOG-reactive Th17 cells produce JAK/STAT activating cytokines. C57BL/6 mice were immunized with MOG and Complete Freund’s Adjuvant and collected after 10 days. Lymphocytes were isolated from mice and cultured under Th17 polarizing conditions (IL-23 (20 ng/mL), IL-1β (5 ng/mL), IL-6 (20 ng/mL), anti-IFNγ (10 μg/mL)) with MOG peptide (35–55) (20 μg/mL) for 4 days. Representative dot plot of RORγT + /IL-17A + CD4 + T cells (**A**). Following polarization, cells were washed and rested for 24 h or restimulated with MOG peptide for 24 h. Supernatants were collected followed by ELISA (**B**). Primary murine astrocytes were transfected with JAK1 siRNA (50 pMols) or a non-targeting (control) siRNA for 48 h. Following transfection cells were cultured with MOG-reactive Th17 cells (2.5 × 10^6^ cells), TNF-α (5 ng/mL), IFNγ (10 ng/mL), or IL-1β (500 pg/mL) for 24 h and gene expression of STAT-dependent genes and JAK1 were measured using RT-qPCR (**C**). UT = untreated. Each data point indicates individual experiments. Statistical significance was determined by T test or 2-way ANOVA (n = 3, *p < 0.05).

To begin to assess the impact of myelin reactive Th17 cells on astrocytes, we first generated MOG-specific T cells by immunizing mice with MOG_35-55_ peptide. Following immunization, splenocyte and lymphocytes were isolated and cultured under Th17 polarizing conditions in the presence of MOG peptide for 4 days. As shown in Fig. 1A, a substantial portion of the CD3 + /CD4 + T cells express RORγT and IL-17, consistent with Th17 polarization. Pathogenic Th17 cells produce GM-CSF, TNF-α, and IFN-γ in addition to IL-17, IL-6, IL-21, and IL-22^23–25,61^. To verify the cultured Th17 cells were antigen reactive and of a pathogenic phenotype following polarization, the cells were either rested or restimulated with MOG peptide for 24 h. The production of IL-17, GM-CSF, TNF-α, IFN-γ, and IL-6 was measured by ELISA and confirmed the cells were consistent with a pathogenic Th17 phenotype (Fig. 1B).

Notably, the Th17 cells were producing IFN-γ, IL-6, and are known to produce IL-21 and IL-22. Following receptor ligation, these cytokines require JAK1 for intracellular signaling^62^, suggesting this may be a key kinase driving the astrocytic response to Th17 cells. To test if the Th17 cells were activating JAK1-dependent signaling in astrocytes, we knocked down JAK1 and cultured the astrocytes with and without pathogenic Th17 cells or treated with TNF-α, IFN-γ, or IL-1β for 24 h. In response to the Th17 cells, gene expression of the JAK/STAT-dependent genes class II transactivator (*Ciita*) and *Cxcl10* was increased (Fig. 1C). Knockdown of JAK1 significantly attenuated expression of *Ciita* and *Cxcl10*, indicating that Th17 cells activate JAK1-dependent gene expression in astrocytes (Fig. 1C).

### JAK1 drives Th17-dependent reprogramming of astrocytes

**Figure 2:**
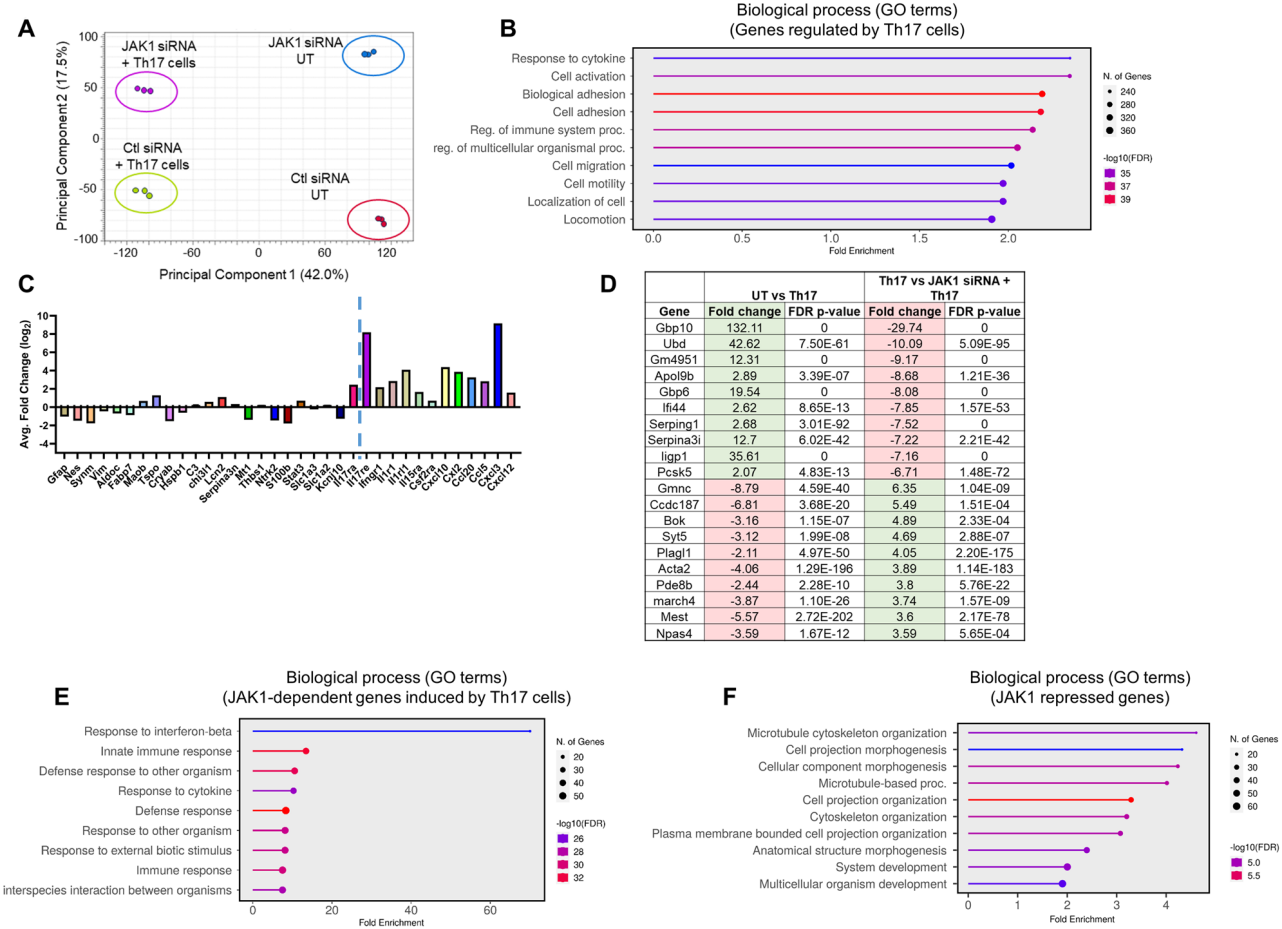
JAK1 drives Th17-dependent reprogramming of astrocytes. Astrocytes were transfected and co-cultured with Th17 cells, as in Fig. 1. Following 24 h of exposure to Th17 cells, astrocyte RNA was collected for RNAseq. Principal component analysis (PCA) of treatment groups. Each data point indicates individual experiments (**A**). Gene ontology based on biological process of gene regulated by Th17 cells (**B**). Average log2 fold change of the indicated genes extracted from the RNAseq dataset, ctl siRNA UT vs. ctl siRNA Th17 cells (**C**). The top 10 genes up and down regulated by Th17 cells in a JAK1-dependent fashion (**D**). Gene ontology is based on biological processes of genes regulated by Th17 cells and dependent on JAK1 (**E**). Gene ontology is based on biological processes of genes repressed by JAK1 in astrocytes co-cultured with Th17 cells (**F**). Ctl = control, UT = untreated. Statistical significance of differential gene expression as determined by Baggerley’s test (n = 3, FDR p < 0.05).

To further examine the impact of Th17 cells on astrocytes, we used bulk RNAseq. As shown in Fig. 2A, principal component analysis (PCA) indicated that both Th17 cells and JAK1 knockdown substantially altered the astrocyte transcriptome. Gene ontology analysis of genes induced in astrocytes by Th17 identified several enriched pathways associated with immune activation and cytokine signaling, as expected (Fig. 2B). Next, we manually curated genes reported to be associated with astrocyte activation^63^. Unexpectedly, most of these genes were unaffected or decreased at this timepoint, including *Gfap* **(**Fig. 2C**,** left of dashed line). In contrast, we observed that several cytokine receptors and chemokines were increased in the astrocytes following the Th17 co-culture (Fig. 2C**,** right of dashed line). We next assessed the genes that were induced by Th17 cells in a JAK1-dependent fashion. Comparing astrocytes without or with JAK1 knockdown and cultured with Th17 cells, there were 97 genes significantly increased 2-fold or more by Th17 cells and decreased 1.5-fold or more by JAK1 knockdown. The top 20 JAK1 regulated genes are shown in Fig. 2D and full gene list in Sup. Fig. 1. The genes increased in a JAK1-dependent fashion in response to Th17 cells were strongly associated with IFN signaling (Fig. 2E). There was a subset of astrocyte genes that were repressed by JAK1 under Th17 co-culture conditions. These genes were primarily associated with pathways regulating cellular morphology (Fig. 2F). These data demonstrate that JAK1 regulates the astrocytic response to Th17 cells in part by driving an interferon response.

### JAK1 regulates astrocytic cytokine receptor expression

**Figure 3:**
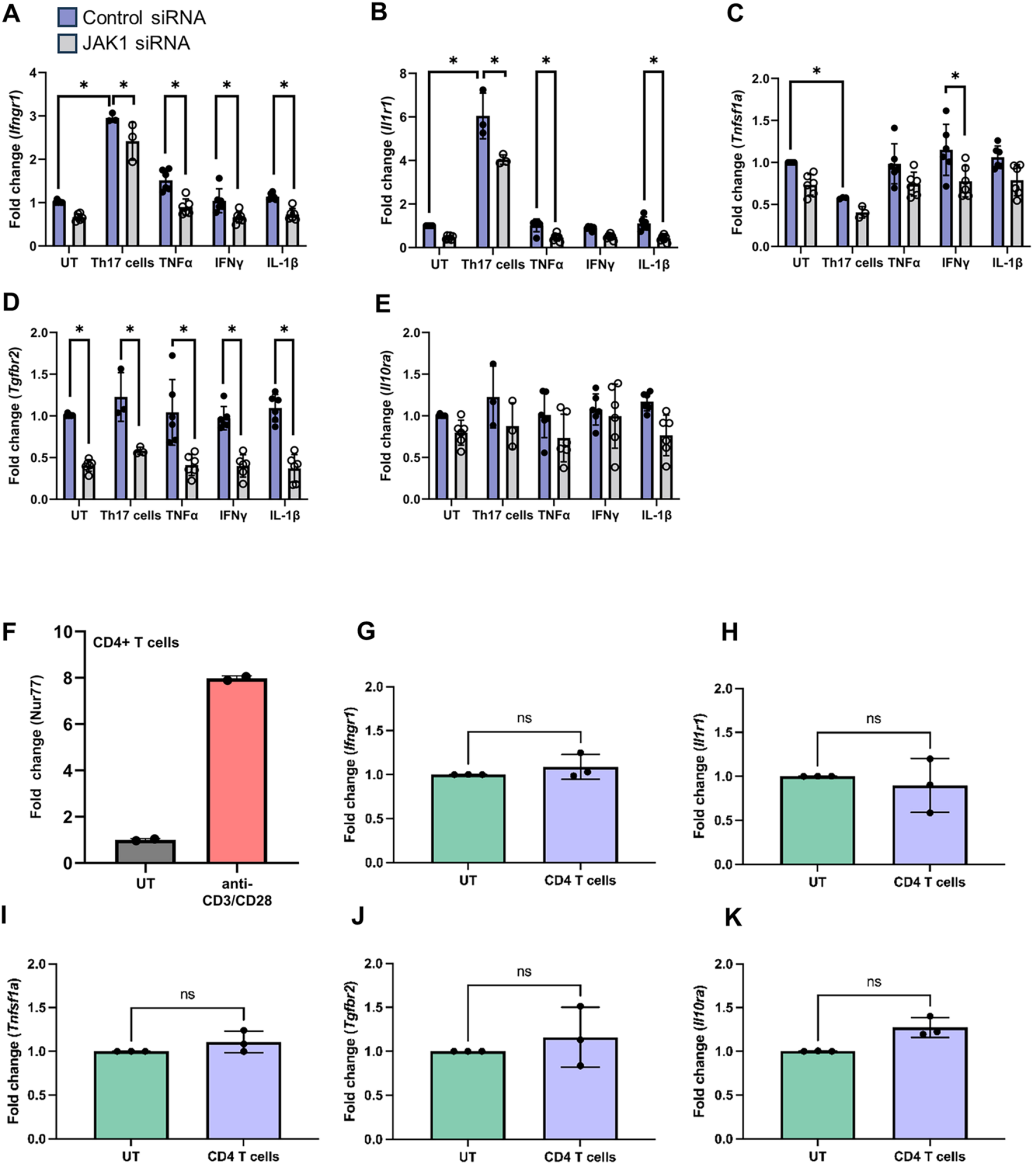
JAK1 regulates astrocytic cytokine receptor expression. MOG-primed Th17 cells were generated as in Fig. 1. Astrocytes without or with JAK1 knockdown were cultured with Th17 cells (2.5 × 10^6^ cells), TNF-α (5 ng/mL), IFN-γ (10 ng/mL), or IL-1β (500 pg/mL). The astrocyte RNA was isolated and gene expression of cytokine receptors was measured using qRT-PCR (**A**–**E**). Astrocytes were cultured with CD4 + T cells that were activated using CD3/CD28 beads for 24 h (**F**) followed by measurement of astrocytic gene expression (**G**–**K**). Data points in F are technical replicates, all other data points indicate individual experiments. Statistical significance was determined by 2-way ANOVA (**A**–**E**) or T test (**F**–**J**) (N = 3–6, *p < 0.05).

The RNAseq data indicated that both Th17 cells and JAK1 could influence cytokine receptor expression in astrocytes. To further examine this, we tested if cytokine receptor gene expression was JAK1 dependent since many pro-inflammatory cytokines are driven by this signaling pathway. We knocked down JAK1 in astrocytes and stimulated as in Fig. 1B. Astrocytic *Ifngr1* and *Il1r1* gene expression was induced by Th17 cells and partially dependent on JAK1 (Fig. 3A,B). Interestingly, *Tnfsf1a* (TNFR1) expression was downregulated in astrocytes by Th17 cells (Fig. 3C). We also examined the anti-inflammatory cytokine receptors, *Tgfbr2* and *Il10ra*. Although *Tgfbr2* and *Il10ra* were not impacted by Th17 cells or cytokine treatment, we identified that basal expression of *Tgfbr2* requires JAK1 (Fig. 3D,E). Additionally, as shown in Fig. 2C, IL17ra and IL17rc are increased in astrocytes by Th17 cells; however, this was independent of JAK1 (Sup. Fig. 1). To test if these effects were specific to Th17 cells, we cultured astrocytes with CD4 + T cells that were activated using bead-bound anti-CD3/CD28 in the absence of polarizing cytokines. To confirm that these T cells were activated, we measured the expression of *Nur77* (nuclear receptor subfamily 4 group A member 1, *Nr4a1*) as a selective marker of T cell receptor stimulation^64^ (Fig. 3F). Activated CD4 + T cells did not have a significant effect on astrocytic cytokine receptor gene expression (Fig. 3G–K). These data suggest that Th17 cells have a unique effect on astrocytic cytokine receptor gene expression.

### Encephalitic Th17 cells modulate cytokine receptor expression in vivo

**Figure 4:**
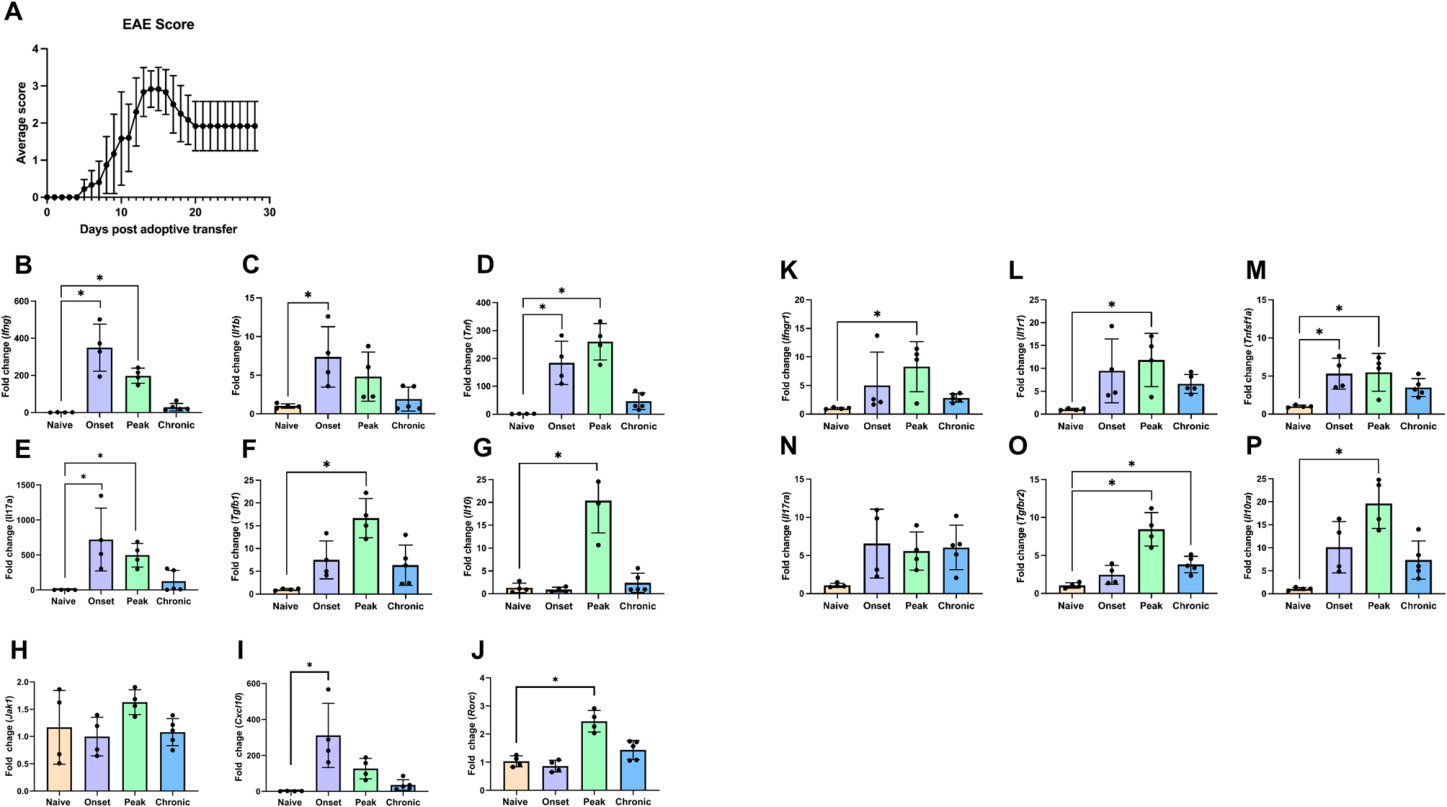
Cytokine and cognate receptor gene expression in the spinal cord during EAE. Th17 adoptive transfer EAE was induced in female wild type C57BL/6 mice. Animals developed typical ascending paralysis over the course of 28 days (**A**). Gene expression in spinal cord samples from naïve, onset, peak, and chronic EAE animals was quantified by qRT-PCR (**B**–**P**). Statistical significance was determined by one-way ANOVA (n = 4–5, *p < 0.05).

To examine cytokine and cytokine receptor gene expression in response to MOG-reactive Th17 cells in vivo, we used the adoptive transfer model of EAE. Disease followed a typical course with animals developing disease by day 6—10 with physical impairment reaching a peak around day 15 followed by modestly diminished severity that was maintained through the chronic phase (Fig. 4A). Gene expression of pro- and anti-inflammatory cytokines from whole spinal cord samples was quantified using qRT-PCR. There was a significant increase in *Ifng, Il1b, Tnf, and IL17a* gene expression at onset and/or peak when compared to the naïve group (Fig. 4B–E). *Tgfb1* and *Il10* gene expression was increased during peak disease (Fig. 4F,G). *Jak1* levels did not change while *Cxcl10* increased, suggesting JAK activation (Fig. 4H–I). This is consistent with previous studies showing JAK/STAT activation during EAE^11,65^. Additionally, *Rorc* (RORγT) levels were elevated even when analyzing this bulk tissue (Fig. 4J). Together with increased *Il17a*, this suggests that at least a portion of the infiltrating Th17 cells maintain their phenotypic polarization after CNS entry. However, this does not exclude the possibility that some of the Th17 cells converted to Th1-like cells as has been shown previously^66,67^. It is notable that expression of all cytokines decreased during the chronic phase of disease with *Ifng*, *Il1b*, and *Il10* returning to near basal levels. These data are consistent with previous studies demonstrating elevated pro- and anti-inflammatory gene expression in the CNS during EAE^68^.

Cytokines drive biological and pathological effects through receptor-mediated signaling. Therefore, we determined the cytokine receptor gene expression from spinal cord samples during EAE. *Ifngr1* was significantly elevated at peak of disease (Fig. 4K). Both *Il1r1*and *Tnfsf1a* had significant increases during peak of disease, and *Tnfsf1a* gene expression was significantly higher at the onset of disease compared to naïve (Fig. 4L–M). Moreover, *Il17ra* appeared increased but did not reach a statistically significant threshold (p = 0.053 at onset) (Fig. 4N). *Tgfbr2* and *Il10ra* receptor both had significant increases at the peak of disease when compared to naïve (Fig. 4O–P). Moreover, *Tgfbr2* was also significantly increased during the chronic phase of EAE (Fig. 4O). These data indicate that expression of key cytokine receptors is elevated during EAE. However, this does not distinguish the contribution of infiltrating immune cells from CNS resident cells, nor if these effects are occurring at the protein level.

### Astrocytes express variable levels of cytokine receptors across the CNS

**Figure 5:**
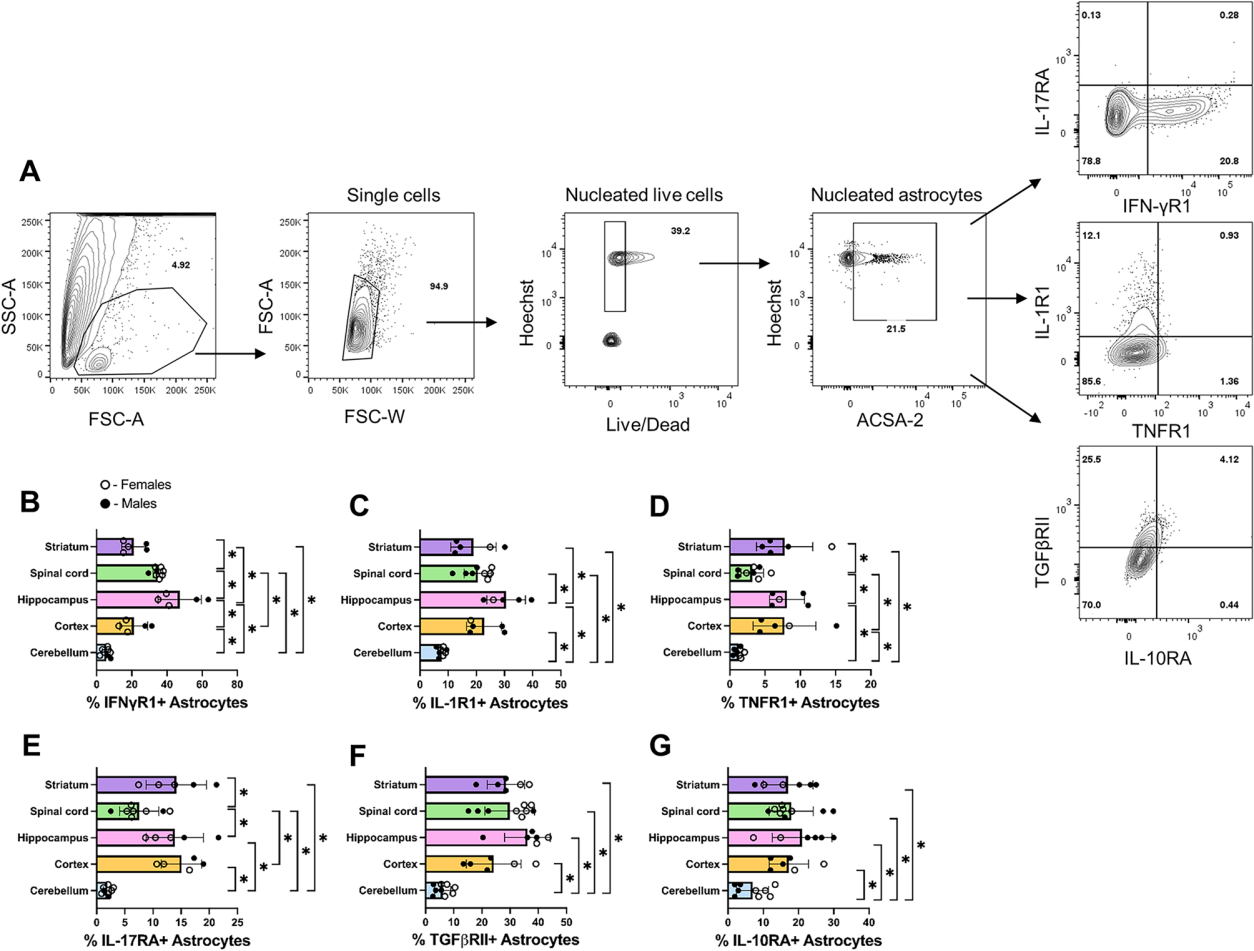
Regional expression of cytokine receptor positive astrocytes in naïve mice. Brains and spinal cords were isolated from naïve C57BL/6 mice. Cells from the indicated region of the CNS were stained with Live/Dead, Hoechst, anti-ACSA-2, and anti-TGFβRII, IL-10RA, IL-17RA, IFNγR1, IL-1R1, or TNFR1. Representative dot plots of gating strategy and frequency of receptor positive astrocytes in the spinal cord (**A**). The percentage of cytokine receptor expressing astrocytes were quantified by flow cytometry (**B**–**G**). Statistical significance was determined by one-way ANOVA (n = 6–9, p < 0.05).

Based on T cell—astrocyte co-cultures and bulk RNAseq data, we hypothesized that autoreactive Th17 cells alter cytokine receptor expression on astrocytes in vivo. Before testing this, we characterized the basal protein levels and regional distribution of astrocytes possessing these cytokine receptors on the cell surface by flow cytometry. Astrocytes were labeled and identified as live, nucleated, ACSA-2 positive single cells, and analyzed for cytokine receptor expression. (Fig. 5A). Astrocytes were analyzed from the cerebellum, cortex, hippocampus, spinal cord, and striatum. IFNγR1-positive astrocytes were significantly increased in the hippocampus compared to the other four regions of the CNS (Fig. 5B). IFNγR1-positive astrocytes were also significantly increased in the spinal cord compared to the cerebellum, cortex, and striatum. IL-1R1 had a similar pattern in which the hippocampus was significantly increased compared to the cerebellum, spinal cord, and striatum (Fig. 5C). TNFR1 is significantly lower in the spinal cord and cerebellum compared to the other observed regions of the CNS (Fig. 5D). We observed a significantly higher percentage of IL-17RA-positive astrocytes in the striatum, hippocampus, and cortex compared to the lower percentage within the spinal cord and the cerebellum (Fig. 5E). The percentage of TGFβRII and IL-10RA-positive astrocytes were significantly higher in the striatum, spinal cord, hippocampus, and cortex compared to the cerebellum (Fig. 5F,G). Collectively, the cytokine receptors showed similar patterns of expression, with the striatum, spinal cord, hippocampus, and cortex having the highest percentage of positive astrocytes and cerebellum having the lowest percentage of receptor positive astrocytes.

### Cytokine receptors exhibit dynamic expression on astrocytes during EAE

**Figure 6:**
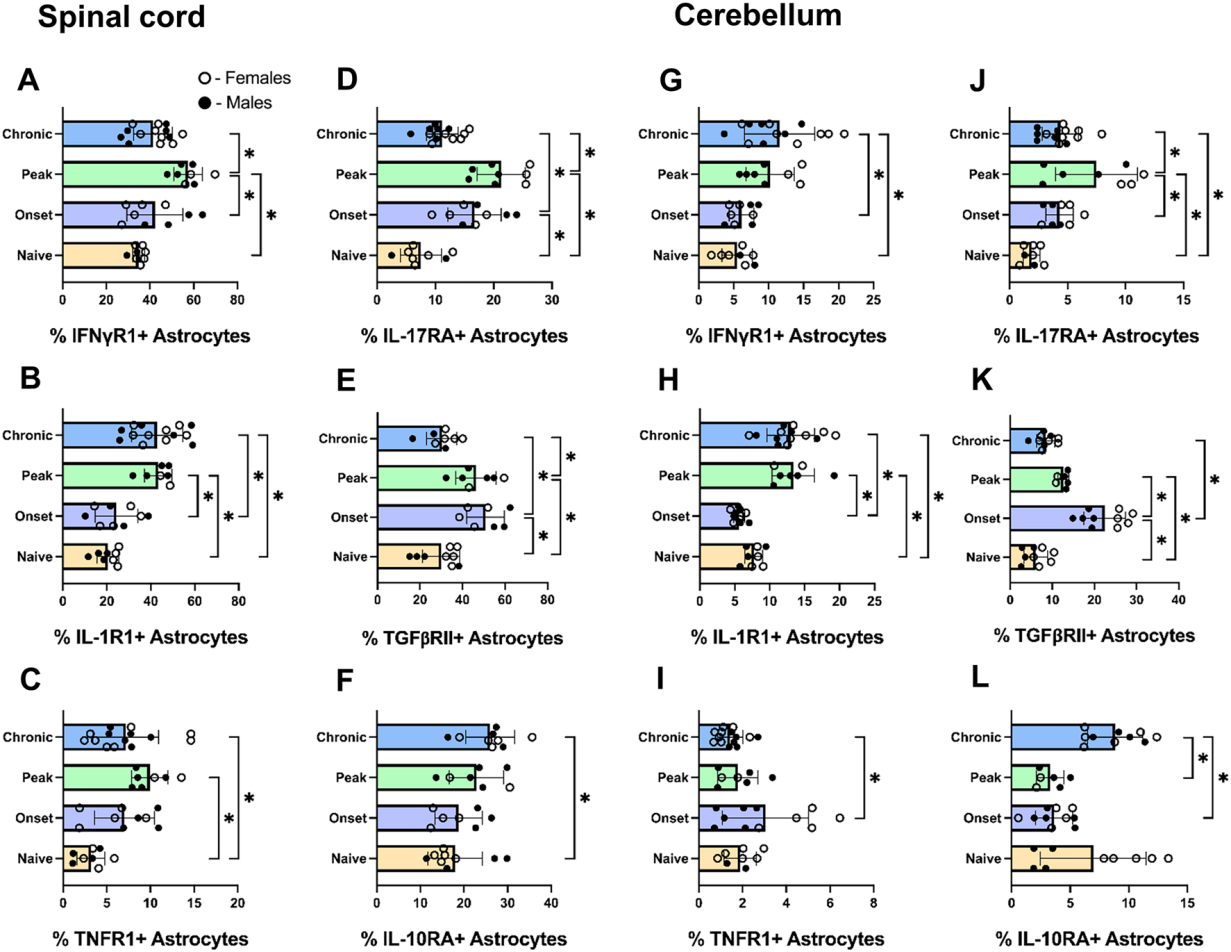
Modulation of cytokine receptor positive astrocytes during adoptive transfer EAE. Th17 adoptive transfer EAE was induced in wild type C57BL/6 mice. The spinal cord (**A**–**F**) and cerebellum (**G**–**L**) were isolated from each animal and live, nucleated astrocytes expressing IFNγR1 (**A, G**), IL-1R1 (**B, H**), TNFR1 (**C, I**), IL-17RA (**D, J**), TGFβRII (**E, K**), or IL-10RA (**F, L**) were quantified flow cytometry and compared to the naïve samples from Fig. 5. Statistical analysis was determined by one-way ANOVA (n = 6–14, p < 0.05).

The cerebellum and spinal cord are the regions of the CNS most affected in EAE^69^. After determining basal levels of cell surface cytokine receptor expression on astrocytes we examined dynamic expression during the course of Th17 adoptive transfer EAE. We induced adoptive transfer EAE as described in Fig. 4 and then measured receptor positive astrocytes at 3 time points: onset, peak, and chronic phases of disease. These were compared to the naïve animals from the analysis shown in Fig. 5. Significant changes in cytokine receptor positive astrocytes were observed in the cerebellum and spinal cord between the three phases of disease. There is a significant increase in IFNγR1-positive astrocytes in the spinal cord at the peak phase of disease followed by a significant decrease at the chronic phase in which the receptor level appears to return to near naïve level (Fig. 6A). IL-1R1-positive astrocytes significantly increase at the peak of disease and remain elevated into the chronic phase of disease (Fig. 6B). TNFR1-positive astrocytes increase at the peak of disease (Fig. 6C). The percent of IL-17RA-positive astrocytes increases at the onset and peak of disease followed by a significant decrease in the chronic phase (Fig. 6D). In the spinal cord the anti-inflammatory TGFβRII shows a similar trend as IL-17RA-positive astrocytes increasing at onset and peak and returning to basal levels at the chronic phase (Fig. 6E). The anti-inflammatory IL-10RA-positive astrocytes only increase at the chronic phase of disease (Fig. 6F).

Within the cerebellum IFNγR1 showed a gradual increase from onset to chronic phase of disease at which it was significantly elevated (Fig. 6G). Similar to the trend in the spinal cord, cerebellar IL-1R1-positive astrocytes increase at peak and maintain elevated levels of the receptor through the chronic phase of disease (Fig. 6H). Although not significant, TNFR1 trends toward an increase during the onset of EAE and remains significantly lower in the chronic phase compared to onset levels (Fig. 6I). IL-17RA-positive astrocytes increase at the peak of disease and then decrease significantly at the chronic phase (Fig. 6J). TGFβRII displays a significantly elevated level at onset compared to naïve TGFβRII-positive astrocytes followed by significant decrease at peak and a return to basal levels at the chronic phase of disease (Fig. 6K). The percentage of IL-10RA-positive astrocytes decreased slightly at onset and peak, and then significantly increased during the chronic phase of disease (Fig. 6L). Taken together, the spinal cord and cerebellum display dynamic levels of cytokine receptors on the cell surface of astrocytes and suggest the existence of both pro- and anti-inflammatory subpopulations of astrocytes that may be influenced by the effector molecules of infiltrating Th17 cells.

## Discussion

There is an abundance of evidence that infiltrating immune cells, particularly Th17 cells, reprogram astrocytes in neuroinflammatory conditions. Encephalitogenic Th17 cells produce soluble mediators that regulate astrocytic homeostatic functions as well as influence production of cytokines, chemokines, and neurotrophic factors^13,14,70^. In MS and EAE, pathogenic T cells can activate astrocytes leading to increased proliferation and formation of glial borders (aka, scar)^13^. These modified astrocytes produce cytokines and chemokines to recruit peripheral immune cells to the CNS^16,71^. Autoreactive Th17 cells produce several neuroinflammatory cytokines including IFN-γ and IL-6, which utilize the JAK1/STAT1/3 signaling pathway, suggesting JAK1 may be an important kinase in astrocytic responses to Th17 cells. In this study we have identified that the astrocytic response to myelin reactive Th17 cells is mediated in part through JAK1 dependent signaling. JAK1 was largely responsible for imposing an IFN responsive gene expression signature in astrocytes. Of the genes significantly changed in response to Th17 cells JAK1 regulated 13.5%. This is consistent with Th17 cells also producing cytokines that signal independent of JAK1, such as GM-CSF and TNF-α. Previous work identified that GM-CSF signaling in astrocytes drives MAFG expression contributing to EAE pathology by promoting an inflammatory program^72^. This implies that JAK2, as a request kinase for GM-CSF signaling^73^, also drives astrocyte gene expression that can contribute to EAE pathology. Additionally, TNF-α and other pro-inflammatory cytokines signal through NF-κB, and inhibition of this pathway in astrocytes attenuates EAE^74^. Overall, autoreactive T cells engage a multitude of intracellular signaling networks to drive transcriptional and metabolic changes in astrocytes that contribute to CNS inflammation and functional outcomes^13,70,75^.

The JAK/STAT pathway is known to play a crucial role in neuroinflammation mediating the response of several key cytokines in EAE^76,77^. Targeting JAK/STAT signaling using small molecule inhibitors blocks the effects of key cytokines such as IL-6, IL-12, IL-23, GM-CSF, and IFN-γ that are involved in EAE and MS^77^. The JAK1/JAK2 inhibitor, AZD1480, inhibits differentiation of Th1 and Th17 cells, and is protective in several models of EAE^11^. Importantly, however, is that context dictates the effects of JAK/STAT signaling. Previous work has shown that STAT3 drives astrogliosis and glial border formation following spinal cord injury, which was vital to constrain inflammation and preserve motor function^78^.

Astrocytes were previously thought to be a mostly homogenous cell type; however, recent studies have shown subpopulations of astrocytes exist^33,40^. Transcriptomic profiling has revealed heterogeneity through gene signatures across brain regions and in region-specific astrocyte transcriptomes^40–47,63,79^. Astrocyte diversity is influenced by transcriptional regulation^46,48^. Additionally, there is a host of transcriptional regulators that drive astrocytic gene expression in models of CNS diseases and neuroinflammation^48^. Our data suggest astrocytes differentially express cytokine receptors based on CNS region. As with previous studies, it is unclear if this reflects a different developmental program or if this is reflective of differential gene expression driven by local stimuli^75^. The distribution of cytokine receptor expressing astrocytes may be indicative of the cytokine producing cells they are most likely to encounter in their microenvironment. As shown previously, astrocytes enriched in interferon stimulated genes (ISGs) and expressing high levels of CXCL10 reside near CNS borders where this astrocytic phenotype was postulated to be induced through interactions with lymphocytes^47^. In line with this concept, we identified that Th17 cells robustly induce an IFN signature, including CXCL10, through a JAK1 dependent mechanism. In addition to the genes shown in Fig. 2, Th17 cells also increased the expression of TRAIL and PD-L1 in astrocytes. Although TRAIL was excluded from our initial analysis because it was below our basal expression cutoff. Astrocyte-derived TRAIL and PD-L1 have been shown to drive T cell apoptosis and suppression^75,80^. This suggests that although Th17 cells strongly induced lymphocyte and monocyte recruiting chemokines in astrocytes, they also induce suppressive molecules which may temper inflammation.

Using adoptive transfer EAE, we identified that Th17 cells influence the cytokine receptor profile on astrocytes, suggesting that these T cells can alter the astrocytic responsiveness to key cytokines. Taken together, our co-culture and EAE experiments indicate that Th17 cells directly induce the expression of IFNγR1 and IL-1R1 on astrocytes, while TNFR1, TGFβRII, and IL-10RA are likely regulated through an indirect mechanism. TGFβRII positive astrocytes increased at the onset of disease, remained elevated at the peak of disease, and returned to basal levels at the chronic phase of disease. IL-10RA positive astrocytes are increased during the chronic phase. Furthermore, we observed a subset of astrocytes that were double positive for TGFβRII and IL-10RA in the spinal cord and cerebellum (Sup. Fig. 2). This may suggest the existence of a subset of astrocytes that have anti-inflammatory properties and that their increase during disease contributes to the transition to the chronic phase of EAE in which there is less inflammation.

While our work contributes to understanding the interaction between astrocytes and T cells, there are a few limitations. Notably, the Th17/astrocyte co-cultures did not use a transwell to allow for physical interaction between the cells. Despite washing away the T cells, there were obvious T cell-derived transcripts in the RNAseq data. We minimized this effect in the analysis by filtering transcripts that were not present (RPKM < 1) in the untreated astrocytes. From this, some genuine astrocyte genes that are very lowly expressed under basal conditions may have been omitted. Neonatal astrocytes were cultured with serum (which is also required for T cells) and are more progenitor-like, which may not faithfully represent mature astrocytes in vivo^81^. This culture method is a potential limitation; however, this approach allows evaluation of the direct effects of T cells on astrocytes, and we identified similar effects on cytokine receptor expression in vivo. While astrocytes have been analyzed by FACS and flow cytometry in many previous studies^72,82–86^, this could represent a limitation in our study. These cells are highly complex with many branches. The physical distribution of CNS tissues required for flow cytometry is likely to rupture the membrane of many astrocytes. We only analyzed cells that were nucleated and had intact plasma membranes. Our analysis may be biased to astrocytes that are less complex and therefore more likely to remain intact during tissue processing.

Despite these modest limitations, this study demonstrates that autoreactive Th17 cells drive widescale gene expression changes in astrocytes through JAK1-dependent and independent mechanisms. Among these genes were several vital cytokine receptors that vary depending on CNS region and are dynamically regulated during EAE. Collectively, this suggests that astrocytic cytokine responsiveness is plastic and modified by interactions with immune cells.

## Materials and methods

### Mice

C57BL/6 J mice (Jackson Laboratories) were housed in the vivarium under the care of the Office of Lab Animal Resources. Mice were housed at 21 °C on a 12 h light–dark cycle with free access to food and water. All procedures were consistent with the National Institutes of Health Guide for the Care and Use of Laboratory Animals. The study was approved by the institutional animal care and use committee.

### T cell polarization

C57BL/6 mice were immunized in each hind flank with 100 μl of an emulsion containing MOG_35—55_ peptide (1 mg/mL) and Complete Freund’s Adjuvant (CFA) (1.5 mg/ml heat killed mycobacterium tuberculosis) and lymphocytes and splenocytes collected after 10 days. Cells were made into a single cell suspension by pushing the tissue through a 100 μm cell strainer followed by red blood cell lysis using Ammonium-Chloride-Potassium (ACK) buffer for 5 min at 37 °C. Cells were cultured for 4 days in RPMI 1640 with 10% FBS, 100 U/mL penicillin, 100 μg/mL streptomycin, 1 × nonessential amino acids, 1 mM sodium pyruvate, 2.5 μM 2-mercaptoethanol, and 2 mM L-glutamine. Cells were polarized by the addition of IL-23 (20 ng/mL), IL-1β (5 ng/mL), IL-6 (20 ng/mL), anti-IFNγ (10 μg/mL), and MOG_35–55_ (20 μg/mL). Polarization was confirmed by flow cytometry. After four days of polarization, cells were washed and stimulated with Phorbol 12-myristate 13-acetate (PMA) (25 ng/mL) and ionomycin (1 ug/mL) followed by treatment with Brefeldin A (10 ug/mL) for 4.5 h. Cells were washed with PBS and stained with Live/Dead violet for 20 min at 4 °C. Cells were washed and incubated in FACS buffer (1%BSA/PBS) with a neutralizing anti-Fc receptor antibody for 10 min, followed by the addition of anti-CD3-PECy5 and anti-CD4-BV421 and incubated for 30 min at 4 °C. Cells were washed with permeabilization buffer and fixed with fixation/permeabilization solution (Invitrogen) for 20 min at 4 °C. Cells were washed with permeabilization buffer followed by intracellular staining with anti-IL17A-eflour506 and anti-RORγt-PerCP-eflour710 for 45 min at 4 °C. Cells were washed and analyzed using Cytek Aurora flow cytometry and analyzed with FlowJo v10.10.0 Software.

### ELISA

Cytokine concentrations were determined from 100 μl of cell culture supernatant using Luminex multiplex ELISA or sandwich ELISA, according to the manufacturer’s protocol.

### Astrocyte-Th17 cell co-culture

Primary astrocytes were isolated from P0-P2 pups as previously described^87^. Astrocytes were then cultured in DMEM with 10% FBS, 16 mM HEPES, 1 × nonessential amino acids, 2 mM L-glutamine, 100 units/ml penicillin, 100 µg/ml streptomycin, and 50 µg/ml gentamicin. Astrocytes were separated from microglia by shaking at 200 rpm for 2 h followed by trypsinization and plating into 6-well plates. T cells were cultured and polarized as described above. After 4 days polarization, CD4 + T cells were isolated by buoyancy activated cell sorting (Akadeum) then added to astrocyte cultures.

### Adoptive transfer EAE model

Female mice were MOG immunized followed by lymphocyte and splenocyte isolation and polarization as above. Male and female recipient mice on a C57BL/6 background (aged 3–4 months) were given intraperitoneal injections of 5 × 10^6^ cells per mouse. Recipient mice developed EAE and were collected at various time points during the onset (5–8 days), peak (13–15 days), and chronic (28 days) phases of disease. Assessment of EAE was as follows: 0, no disease; 1, decreased tail tone; 2, hind limb weakness or partial paralysis; 3, complete hind limb paralysis; 4, front and hind limb paralysis; and 5, moribund state^7,8^.

### Isolation of CNS tissue and flow cytometry

Tissue dissociation for flow cytometry was adapted from^83,88^. Mice were deeply anesthetized and perfused with 20 mL of DPBS. Spinal cords and brains were isolated. Brains were sectioned into coronal slices and the cortex, hippocampus, cerebellum, and striatum were isolated. The number of animals differs between regions because in some animals only the spinal cord and cerebellum were analyzed. Separated tissue was minced and placed in 1 mL of accutase for 40 min at 4 °C. Tissue was resuspended in Hibernate A medium (Brain Bits, LLC) and transferred to a 2 mL Dounce homogenizer and tissue was disrupted until no large pieces remained. Cell suspension was filtered using a 100 μm cell strainer. Isolated cells were stained for flow cytometry (BD Aria) for the following markers: Hoechst, Live/Dead stain, FITC anti-mouse ACSA-2, APC TGFBRII, APC TNFR, APC IL17RA, PE IFNGR1, PE IL1R1, and PE IL10RA. An unstained control and single stain control for each fluorophore were used to identify the cell population of interest, set flow cytometer voltage, and calculate compensation. Flow cytometry data was analyzed using FCS Express 6 or FlowJo.

### Quantitative real-time PCR analysis

RNA was isolated using 1 ml of TRIzol according to the manufacture's protocol. RNA was quantified using a NanoDrop system (Fisher). One microgram of RNA was used for cDNA synthesis. RNA was mixed with 0.5 μg of oligo dT primer and incubated at 70 °C for 5 min followed immediately by 5 min on ice. A mix containing reaction buffer, Moloney murine leukemia virus reverse transcriptase, deoxynucleotide triphosphates, and RNasin was added and incubated at 42 °C for 60 min. The reaction was terminated by incubation at 95 °C for 5 min. The cDNA was then analyzed by qPCR performed using probe-based gene expression assays using Quant studio 3 qPCR machine. Reactions were analyzed using the ΔΔCt method.

### RNA sequencing and bioinformatics

RNA was isolated using TRIzol, as above. DNase treatment, library preparation, and sequencing were conducted by Admera Health. Briefly, Ribozero Plus + NEBNext Ultra II Directional was used to generate libraries. Reads (2 × 150) were collected on an Illumina sequencer at 60–90 million reads per sample. The reads were then trimmed, QC, mapped, and analyzed for differential gene expression using CLC Genomics Workbench (Qiagen). To determine Th17 regulated genes (untreated vs. Th17), the data were filtered based on the following criteria, genes with < 1 RPKM in the untreated group were removed, genes with FDR < 0.05 and fold change absolute value > 2 were used in subsequent analyses. The Th17 regulated genes with an absolute fold change of > 1.5 fold in response to JAK1 knockdown were identified as JAK1 dependent (Th17 vs. JAK1 siRNA Th17). Gene ontology was analyzed using ShinyGO v0.77 http://bioinformatics.sdstate.edu/go/^89^.

### Statistical tests

All experiments were done at least 3 independent times. A priori sample size calculation was not performed for this study. Experiments involving mice consisted of single experimental groups, therefore blinding and randomization were not applicable. Each data point (n) on the graphs indicates an independent experiment or animal, bar graphs indicate the average and error bars are standard deviation. Students T test, one-way ANOVA with post-hoc Tukey test, and two-way ANOVA with post-hoc Tukey test were conducted using GraphPad Prism software. The level of statistical significance is defined as p < 0.05.

### Arrive guidelines

The findings in this study are reported in accordance with ARRIVE guidelines.

## Data availability

Data and related materials available on request. RNA sequencing data is available in the Gene Expression Omnibus under accession number GSE262540.

### Supplementary Information

Supplementary Figures.

## Abbreviations

ACSA-2: Astrocyte cell surface antigen-2
BBB: Blood brain barrier
CCR: Chemokine receptor
CD: Cluster of differentiation
CNS: Central nervous system
CXCL: C-X-C motif chemokine ligand
EAE: Experimental autoimmune encephalomyelitis
ELISA: Enzyme-linked immunosorbent assay
GM-CSF: Granulocyte–macrophage colony-stimulating factor
ICAM1: Intracellular adhesion molecule-1
IFN-γ: Interferon-gamma
IFNγR1: Interferon-gamma receptor 1
IL-1β: Interleukin-1-beta
IL-1R1: Interleukin-1 receptor 1
IL-6: Interleukin-6
IL-10: Interleukin-10
IL-10RA: Interleukin-10 receptor A
IL-12: Interleukin-12
IL-17: Interleukin-17
IL-17RA: Interleukin-17 receptor A
IL-23: Interleukin-23
JAK: Janus kinase
MOG: Myelin oligodendrocyte glycoprotein
MS: Multiple sclerosis
NF-κB: Nuclear factor kappa B
PCA: Principal component analysis
PD-L1: Programmed death-ligand 1
RORγτ: Retinoic-acid-receptor-related orphan nuclear receptor
STAT: Signal transducer and activator of transcription
TH: T helper
TGFβ: Transforming growth factor-beta
TGFβRII: Transforming growth factor-beta receptor 2
TNF-α: Tumor necrosis factor-alpha
TNFR1: Tumor necrosis factor receptor 1
TRAIL: Tumor necrosis factor-related apoptosis-inducing ligand
VCAM: Vascular cell adhesion molecule
