# Supplementary material for: Myelin oligodendrocyte glycoprotein reactive Th17 cells drive Janus Kinase 1 dependent transcriptional reprogramming in astrocytes and alter cell surface cytokine receptor profiles during experimental autoimmune encephalomyelitis
Source: Sci Rep. 2024 Jun 7;14:13146. doi: 10.1038/s41598-024-63877-0 (PMC11161502; doi:10.1038/s41598-024-63877-0)
Supplement: Supplementary file 1 — Supplementary Figures. [file 41598_2024_63877_MOESM1_ESM.pdf]

Supplemental Figure 1

	Th17 vs Th17 + JAK1 siRNA		UT vs Th17	
Gene	Fold change	FDR p-value	Fold change	FDR p-value
Gbp10	-29.74	0	132.11	0
Ubd	-10.09	5.09E-95	42.62	7.50E-61
Gm4951	-9.17	0	12.31	0
Apol9b	-8.68	1.21E-36	2.89	3.39E-07
Gbp6	-8.08	0	19.54	0
Ifi44	-7.85	1.57E-53	2.62	8.65E-13
Serping1	-7.52	0	2.68	3.01E-92
Serpina3i	-7.22	2.21E-42	12.7	6.02E-42
Iigp1	-7.16	0	35.61	0
Pcsk5	-6.71	1.48E-72	2.07	4.83E-13
Cdhr1	-6.11	2.12E-61	2.44	1.02E-09
C1s1	-5.54	9.38E-264	3.76	2.50E-166
Tgtp1	-5.12	8.92E-259	120.69	0
F830016B0	-5.11	4.93E-222	27.98	0
Gbp9	-4.97	3.36E-229	5.37	2.88E-254
Gbp2	-4.78	2.04E-248	32.52	0
Igtp	-4.48	1.33E-224	29.52	0
Gbp5	-4.46	1.58E-209	35.45	0
BC023105	-4.35	1.13E-84	17.12	5.08E-90
Cxcl12	-4.3	3.76E-78	3.02	6.54E-17
Tacr1	-3.95	3.33E-29	2.67	2.90E-19
Tgtp2	-3.86	2.81E-179	129.01	0
Isg15	-3.66	7.40E-20	2.37	7.50E-08
Gbp4	-3.57	1.26E-156	82.31	0
Ifi47	-3.55	1.96E-152	44.75	0
Gbp3	-3.34	6.67E-136	6.7	4.64E-320
Gm12250	-3.28	6.63E-121	115.66	0
Irgm1	-3.18	4.90E-133	10.56	0
Oasl2	-3.11	9.80E-106	2.83	1.85E-90
C1ra	-3.05	3.10E-114	2.41	4.44E-74
Irgm2	-2.93	1.09E-112	8.81	0
Ptx3	-2.91	3.21E-73	3.66	4.05E-101
Gbp7	-2.9	5.24E-110	6.73	0
Il18bp	-2.77	4.61E-17	8.79	4.25E-61
Cxcl10	-2.75	8.01E-97	20.79	0
Ube2l6	-2.71	2.63E-74	4.77	2.20E-148
Ifi204	-2.71	2.92E-58	2.02	3.67E-31
Bst2	-2.7	7.65E-58	2.59	4.00E-54
Herc6	-2.63	3.72E-75	2.73	7.29E-83
Xaf1	-2.62	8.06E-46	2.35	1.26E-09
Wars	-2.61	3.95E-86	3.21	4.41E-128
Irf7	-2.58	7.78E-49	3.44	4.23E-65
Ifi205	-2.54	3.59E-18	6.87	7.63E-26
Vcam1	-2.46	3.73E-82	3.09	4.21E-130
Pla2g16	-2.44	1.19E-63	2.2	2.33E-42
Arrdc4	-2.44	4.95E-63	2.41	1.43E-56

H2-T23	-2.43	2.80E-77	8.86	0
Lcn2	-2.25	5.60E-68	2.16	3.67E-62
Stat1	-2.25	5.78E-67	9.09	0
Exoc3l4	-2.11	2.60E-13	2.05	2.81E-09
Stat2	-2.08	5.78E-49	3.18	2.23E-119
Rnf213	-2.02	6.29E-51	2.2	4.13E-64
Erap1	-1.99	6.34E-45	2.48	8.92E-78
Nupr1	-1.99	2.72E-29	3.44	3.71E-88
H2-T22	-1.97	2.73E-43	3.16	2.69E-122
H2-Q6	-1.92	1.31E-38	7.08	1.68E-288
Oas1a	-1.91	9.73E-24	2.23	7.78E-12
Mnda	-1.9	1.21E-27	4.25	1.43E-85
H2-Q4	-1.88	5.58E-39	2.51	3.43E-83
Mlkl	-1.84	2.87E-20	8.48	5.04E-58
Tap1	-1.82	4.58E-36	6.26	4.35E-318
Amigo2	-1.81	2.17E-05	3.22	5.98E-16
Trim30a	-1.79	1.80E-30	2.02	7.39E-45
Zbp1	-1.79	1.70E-29	23.55	0
Cxcl5	-1.78	2.57E-23	3.5	7.17E-48
Nod1	-1.77	5.99E-25	2.67	2.17E-70
Slfn8	-1.75	5.42E-21	4.85	5.10E-100
9930111J2	-1.75	1.70E-09	10.69	5.17E-54
Tapbpl	-1.74	1.57E-25	4.69	5.68E-165
Camkk1	-1.73	1.59E-04	2.36	7.79E-06
Parp14	-1.73	5.71E-30	4.26	5.17E-204
Abhd15	-1.71	1.81E-06	2.75	2.19E-08
Tmem140	-1.71	1.30E-08	2.86	5.92E-13
Psmb10	-1.69	1.52E-25	6.57	2.48E-272
Lgi2	-1.67	1.21E-19	2.2	7.72E-34
Parp10	-1.67	1.35E-21	3.31	4.91E-107
Psme1	-1.66	1.84E-24	4.5	2.66E-199
Dtx3l	-1.65	2.24E-24	3.87	3.03E-169
Dhx58	-1.65	2.17E-04	2.79	1.21E-17
Tlr2	-1.63	3.78E-21	2.37	1.57E-63
H2-DMb1	-1.62	9.76E-05	7.18	3.23E-46
Ripk2	-1.6	3.92E-17	2.18	4.54E-40
Glipr2	-1.6	2.90E-21	2.73	1.49E-93
Tapbp	-1.59	4.35E-22	2.28	8.56E-70
Irf1	-1.58	3.86E-21	11.42	0
Uba7	-1.57	1.21E-17	4.17	3.34E-124
Psmb8	-1.57	5.98E-20	7.63	0
9930111J2	-1.57	8.47E-19	11.27	1.31E-276
Stra6	-1.56	2.56E-04	2.16	1.17E-06
St8sia1	-1.55	2.26E-11	4.39	1.48E-30
Parp9	-1.55	3.03E-18	3.61	6.88E-144
Psmb9	-1.55	4.90E-17	9.63	1.01E-310
Trim21	-1.55	3.15E-13	3.16	2.08E-74
Slco2a1	-1.54	1.61E-03	4.43	3.40E-24

Angptl4	-1.54	7.06E-12	2.16	5.93E-09
Oas2	-1.54	0.02	3.15	5.67E-09
Casp4	-1.53	5.49E-10	3.83	1.20E-26

Supplemental Figure 2

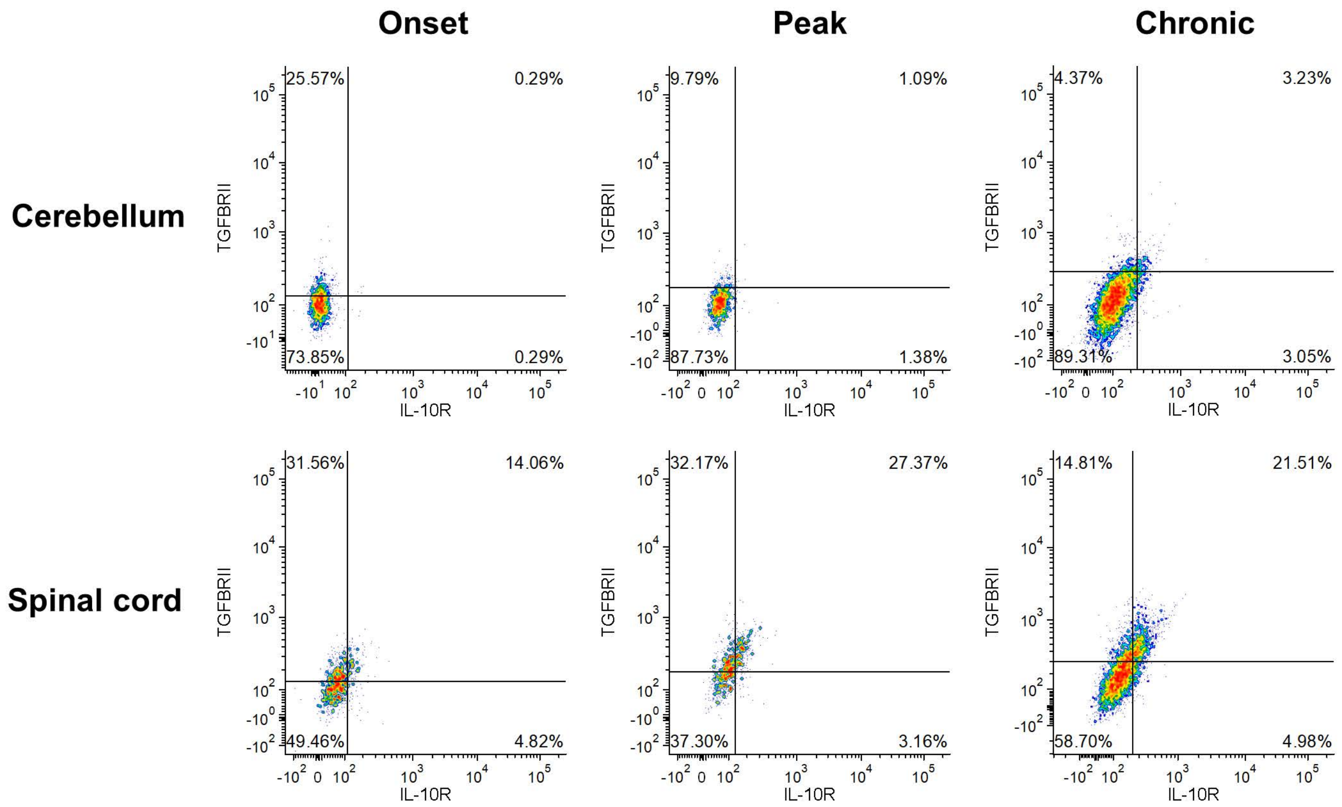

Supplemental Figure Legends

Supplemental Figure 1. Astrocyte genes induced by Th17 cells through a JAK1 dependent mechanism.

Supplemental Figure 2. Representative flow plots of astrocytes from the cerebellum and spinal cord of EAE animals. Astrocytes from onset, peak, chronic stages of EAE were stained for TGFBRII and IL-10R and analyzed based on the gating strategy shown in Figure 5A.
